# Sequence Analysis of a Hybrid HK022/λ Bacteriophage and the Precise Identification of the λ*b515* and λ*b519* Deletion Endpoints

**DOI:** 10.1128/MRA.01118-18

**Published:** 2018-11-08

**Authors:** Rodney A. King, Meghan T. Perez

**Affiliations:** a Department of Biology, Western Kentucky University, Bowling Green, Kentucky, USA; University of Maryland School of Medicine

## Abstract

Bacteriophage O276 is a laboratory-generated hybrid that carries the immunity region of bacteriophage HK022 and all remaining genes from phage λ. Its construction was instrumental in the discovery of RNA-mediated antitermination, an intriguing alternative to the protein-mediated mechanism of transcription antitermination found in most lambdoid phages.

## ANNOUNCEMENT

Most early phage genes in lambdoid phages are located downstream of transcription termination sites, and the suppression of termination, or antitermination, is required for the production of viable phage particles. Early analysis of the immunity region of lambdoid phage HK022 suggested that transcription antitermination occurs but that no phage-encoded antitermination protein is required (1). To confirm that early right-operon transcription is antiterminated in HK022, a hybrid phage (O276) with the immunity region of HK022 and the remainder of its genome from λ was constructed (2). The viability of the hybrid demonstrated that the HK022 immunity region contained functional equivalents of the λ antitermination elements and eventually led to the discovery of specialized RNA molecules that directly modify RNA polymerase and promote terminator readthrough (3).

Bacteriophage λ was isolated by UV induction of a lysogenic strain of Escherichia coli, and HK022 was isolated from sewage as previously described (4, 5). Phage O276 was grown on E. coli MG1655 cells using standard procedures, and the genomic DNA was purified using the phenol-chloroform method (6). Sequencing libraries were prepared using the Ion Shear kit version 2 and the Ion Xpress library kit. Libraries were processed on an Ion Torrent Personal Genome Machine (PGM) using a 314 Chip, and ∼100,000 reads were generated. Single-end reads (100 to 200 bases) were assembled using Newbler version 2.6 with default parameters, yielding a single circular phage contig with at least 120-fold coverage, which was checked for completeness and quality using Consed version 22. The hybrid genome is 41,969 bp long and has a GC content of 50.7%. The hybrid genome is smaller than that of wild-type λ. This difference is primarily due to the *b515* and *b519* deletions that were present in the λ parent. The *b515* deletion removes 1,995 bases, and the *b519* deletion removes 3,177 bases. These deletions were previously characterized; however, no sequence data were provided, and a range of possible endpoints for the *b515* deletion was reported (7, 8). Furthermore, we could not confirm the reported coordinates and size estimate for the *b519* deletion. Our sequencing analysis unambiguously demonstrates the endpoints of both deletions in phage O276. The hybrid phage carries the HK022 *cIts12* mutation (9) that was present in the HK022 parent and a single “G” insertion at position 14,267 that is absent in the published λ genome.

Phage O276 genes were identified using Glimmer (10), GeneMark (11), ARAGORN (12), and tRNAscan-SE (13) and by manual inspection and annotation revision using DNA Master (http://cobamide2.bio.pitt.edu/computer.htm) and PECAAN (https://pecaan.kbrinsgd.org/). Comparison with the published λ and HK022 genomes provided additional verification of the annotations. Gene functions were identified/confirmed using BLASTP (14) and HHPred (15).

Genetic analysis of O276 suggested that the crossovers between the parental phages likely occurred between genes *cII* and *O* in the right operon and between genes *cIII* and IS*903* in the left operon and that the only HK022 genes in O276 (excluding IS*903*) are *nun*, *cI*, *cro*, and *cII* (1). Sequence analysis of the hybrid phage genome confirmed these conclusions.

### Data availability.

The GenBank accession number for *Escherichia* species phage O276 is MH547045. Raw reads are available in the SRA under accession number SRR7774037.
